# Association between maternal depression and emotion and behavior regulation in Peruvian children: A population-based study

**DOI:** 10.1016/j.pmedr.2022.101879

**Published:** 2022-07-01

**Authors:** Akram Hernández-Vásquez, Rodrigo Vargas-Fernández, Fabian Chavez-Ecos, Isabel Mendoza-Correa, José Del-Carmen-Sara

**Affiliations:** a Universidad Nacional Mayor de San Marcos, Lima, Peru; b Centro de Excelencia en Investigaciones Económicas y Sociales en Salud, Vicerrectorado de Investigación, Universidad San Ignacio de Loyola, Lima, Peru; c Universidad Científica del Sur, Lima, Peru; d Sociedad Científica de Estudiantes de Medicina de Ica, Universidad Nacional San Luis Gonzaga, Ica, Peru

**Keywords:** Depression, Child development, Patient health questionnaire, Health surveys, Cross-sectional studies, Peru

## Abstract

Depression is more frequent in women, affecting the early stages of child development. This study aimed to determine the association between maternal depression and self-regulation of emotions and behaviors in Peruvian children under five years. A cross-sectional analytical study of data collected by the 2019 Demographic and Family Health Survey (ENDES) was conducted. The outcome variable was emotion and behavior regulation in children aged 24 to 59 months, and exposure was the presence of depression in women aged 15 to 49 years during the 14 days prior to the survey using the Patient Health Questionnaire (PHQ-9). A generalized linear model of the binomial family was used for reporting crude prevalence ratios and adjusted. The overall prevalence of children who did not self-regulate their emotions and behaviors was 68.8%, while 3.8% of the mothers had moderate depressive symptoms and 2.2% severe symptoms. Regarding the association of interest, moderate and severe depressive symptoms of mothers decreased the probability of children regulating emotions and behaviors in the first model, whereas in the second model, an association was only found with severe depressive symptoms. In conclusion, children of mothers with moderate and severe depressive symptoms had a lower probability of self-regulating their emotions and behaviors. Therefore, it is necessary to develop maternal education, nutritional and social support programs and mental health strategies from the first level of care aimed at reducing social, economic and child factors to reduce the risk of depression in mothers and low early childhood development, which could reduce the risk of developing mental health disorders in adolescence and adulthood.

## 1 Introduction

Maternal depression is a public health problem, which defines a period of persistent sadness or adynamia associated with severe cognitive and somatic symptoms during pregnancy and the postnatal period (Shrivastava et al., 2015). This disorder generates an environment that impedes maternal personal development and child development, resulting in impaired quality of life for mothers and poor cognitive development of children (Slomian et al., 2019). Worldwide, more than 10% of women suffer from maternal depression (Evans et al., 2001), where low- and middle-income countries (LMICs) have almost twice the prevalence compared to high-income countries (Gelaye et al., 2016). Specifically, in LMICs, one in four women have had an episode of depression before childbirth, while one in five women suffered from postpartum depression in 2016 (Gelaye et al., 2016). Despite the fact that the prevalence of maternal depression is high in LMICs, this disorder remains underestimated and with low access to treatment (Oates, 2003). Therefore, health systems should screen for early diagnosis and treatment in order to favor the development of the mother and child.

The higher rates of maternal depression in LMICs compared to high-income countries reflect unfavorable conditions in the woman's environment that contribute to the development of this disorder (Gelaye et al., 2016). Particularly, women residing in LMICs are exposed to intimate partner violence, unwanted pregnancy, lack of social support, low socioeconomic status at the time of pregnancy and lack of emotional support that increase the risk of maternal depression (Gelaye et al., 2016, Fisher et al., 2012). Latin America and the Caribbean is one of the regions with LMICs, where the prevalence of maternal depression ranges between 35 and 50% (Wolf et al., 2002). In this region, the lack of access to culturally competent mental health services means that this maternal disorder persists over time and has consequences for early child development (Alarcón, 2003). In Peru, around 50% of mothers have depression, and only 10% receive treatment for this disorder (Ministerio de Salud, 2018). For this reason, countries with limited resources are at greater risk of having mothers and children with impaired personal and child development, which leads to a greater burden of preventable mental health diseases and an increase in health spending on these pathologies.

In the biomedical literature, it is observed that maternal depression is associated with a deterioration of early child development, especially emotional, behavioral and cognitive (Slomian et al., 2019, Harris and Santos, 2020, Bluett-Duncan et al., 2021, Fatori et al., 2020). In this regard, it is reported that the mother's depressive symptoms are related to an increase in negative emotions and behaviors in the child such as hyperactivity, attention deficit, depression and anxiety, which are accentuated the greater the severity of maternal depression (Fatori et al., 2020). This association starts from the child's neurodevelopment, where neuroplasticity and the formation of neural connections are observed at an early age, contributing to the maturation of the nervous system, the progression of brain functions and the formation of personality (Medina Alva et al., 2015, van der Knaap et al., 2018). In this process, mothers play an essential role in promoting early socioemotional competencies through mother–child interaction in infancy; however, in mothers who suffer from depression from gestation to the postpartum stage, interaction with their children is interrupted, generating alterations in the management of emotions and increased mental health disorders throughout the life of these children (Bernard-Bonnin A-C, Canadian Paediatric Society, Mental Health and Developmental Disabilities Committee, 2004, Urizar and Muñoz, 2021).

In LMICs, mothers present a high prevalence of maternal depression, where the emotional and behavioral development of the child experiences the greatest consequences due to this mental disorder, even more so when women and children are exposed to unfavorable conditions that increase the risk of mental health disorders (Gelaye et al., 2016). Despite this problem, scientific evidence on the association between maternal depression and the emotional and behavioral development of children in LAC countries is still scarce. Therefore, the aim of the present study was to determine the association between maternal depression and the self-regulation of emotions and behaviors in Peruvian children under five years of age.

## 2 Materials and methods

### 2.1 Study design and population

A cross-sectional analytical observational study of the data collected by the 2019 Demographic and Family Health Survey (ENDES - acronym in Spanish) was conducted. The ENDES is a population-based survey conducted annually by the National Institute of Statistics and Informatics (INEI - acronym in Spanish), which aims to provide updated information on the main health indicators in the Peruvian population. This survey applies three questionnaires that collect information on the household and its members, the individual health of the women (applied to all women of childbearing age from 15 to 49 years old) and health (applied to all people over 15 years old). Since 2018, the ENDES has been conducting an annual questionnaire on early childhood development in children aged 9 to 71 months. For detailed information on the methodology of this survey, consult the 2019 ENDES data sheet (Instituto Nacional de Estadística e Informática, 2019).

The 2019 ENDES used a two-stage sampling (clusters and households), which is probabilistic, balanced, stratified and independent. The survey is representative of urban and rural areas throughout Peru, according to geographic domain (coast, highlands and jungle) and for the administrative regions of the country (Instituto Nacional de Estadística e Informática, 2019).

The present study included Peruvian women of childbearing age (15 to 49 years) with complete data from the Health Questionnaire and their minor children between 24 and 59 months of age with complete data from the Early Childhood Development Questionnaire. In a household with two or more children, information of the youngest child was obtained.

### 2.2 Variables and measurements

#### 2.2.1 Outcome

The outcome variable was the regulation of emotions and behaviors of children aged 24 to 59 months. This variable is the ability acquired in infancy to recognize, express and communicate emotions and manage their reactions in different contexts. To construct this variable, the following three questions from the Early Childhood Development Questionnaire, answered by the mother of the child, were taken into account: 1) does (name of the child) cry, scream or throw tantrums most of the time?; 2) when (name of the child) wants something and you tell him/her to wait, does he/she usually wait “quietly”?; and 3) when (name of the child) wants something and you tell him/her no, does he/she usually hurt himself/herself, attack others or damage things? Responses were two-choice: no (value 0) and yes (value 1). An affirmative answer to each of the three questions (3 points) was considered as the child having adequate regulation of emotions and behaviors for their age, while a negative response(s) (0 to 2 points) was considered as inadequate regulation.

These questions are part of an instrument developed and validated by the Ministerio de Desarrollo e Inclusión Social (MIDIS) to assess early child development (ECD) in Peru based on the Emotional Regulation Checklist (ERC) (Ministerio de Desarrollo e Inclusión Social, 2019). The content validity, in a qualitative way, was carried out by experts in each of the ECD results as well as the discussion of the questions with specialists members of the multisectoral commission; quantitatively, the content validity was executed by 20 academic expert judges and national and international professors, being ten judges for result 6 reaching a degree of agreements between judges in the evaluated area with proposed age, calculated with the Aiken V indicator being the value obtained greater than 0.8 (Ministerio de Desarrollo e Inclusión Social, 2019).

#### 2.2.2 Exposure

The exposure variable of the analysis was the presence of depression during the 14 days prior to the survey, measured by the Patient Health Questionnaire (PHQ-9). The PHQ-9 is a depression screening instrument that has been validated in many countries for use in the general population of women and pregnant women, including Peru through expert judgment and had shown optimal values of reliability and validity (Calderón et al., 2012, Zhong et al., 2014, Villarreal-Zegarra et al., 2019).

The PHQ-9 contains nine questions that assess the presence of depressive symptoms. The responses have a severity index, ranging from 0 (not at all) to 3 (almost every day) with the total score ranging from 0 to 27 points. The score is classified into five categories: minimal (0–4), mild (5–9), moderate (10–14), moderately severe (15–19) and severe (20–27) (Kroenke et al., 2001). For the present study, the three categories used in a previously published study were considered, based on the instrument score: 0–9 (mild), 10–14 (moderate) and 15 or more (severe) (Hernández-Vásquez et al., 2020).

#### 2.2.3 Covariates

The study covariates were included according to previous reports in the literature (Hernández-Vásquez et al., 2020, Alarcón-Guevara et al., 2021, Valladares-Garrido et al., 2020, Villarreal-Zegarra et al., 2020, Villarreal-Zegarra and Bernabe-Ortiz, 2020, Ibanez G, Bernard JY, Rondet C, Peyre H, Forhan A, Kaminski M, Saurel-Cubizolles M-J, EDEN Mother-Child Cohort Study Group, 2015, Hoffman et al., 2006, Wu et al., 2020). The main covariates included (values of the variables are indicated in parentheses): the age of the mother (in years), level of education of the mother ([no education/elementary]/high school/college), marital status of the mother (single/cohabitation with partner/[separated/divorced/widowed]), ethnic self-identification of the mother (non-native/native), child sex (female/male), age of the child (in months), undernutrition of the child (no/yes), hemoglobin level of the child (in g/dL), breastfeeding time (in months), desired pregnancy (no/yes), adequate prenatal controls (APCs) (according to the World Health Organization (WHO) which are considered adequate when a pregnant woman has eight or more APCs) (no/yes), birth order number (continuous numerical), wealth quintile (quintile 1[richest]/quintile 2/quintile 3/quintile 4/quintile 5[poorest]), area of residence (urban/rural) and natural region (jungle/highlands/coast).

### 2.3 Statistical analysis

Stata 14 software (Stata Corporation, College Station, Texas, USA) was used to import the databases in .sav format and to perform the statistical analysis. For all analyses, complex sampling characteristics and weighting factors were included using the svy command.

Categorical variables in the study were described as absolute frequencies and weighted proportions, while quantitative variables were expressed as means and their standard deviation. The Chi-square test with Rao-Scott correction (in categorical variables) or the Student’s t-test (in numerical variables) was used to compare the study variables.

The association between maternal depression and emotions and behaviors self-regulation was assessed using a generalized linear model of binomial family with logarithmic link function to report crude prevalence ratios (PR) and adjusted PR (aPR) for the covariates of interest that were included based on the literature. Each estimate was expressed with its respective 95% confidence interval (95% CI). Collinearity among covariates was analyzed previously using the variance inflation factor (VIF), and multicollinearity was considered when the VIF exceeded a value of 10. A p value < 0.05 was considered statistically significant for all statistical tests.

### 2.4 Ethical considerations

The approval of an ethics committee was not requested because this was an analysis of secondary data that is in the public domain and does not allow the identification of the participants evaluated. The databases are available at: http://iinei.inei.gob.pe/microdatos/.

## 3 Results

### 3.1 Sociodemographic characteristics of the mothers

Data from a total of 4738 participants were included in the analysis. The mean age of the mothers was 31.8 years (SD [standard deviation]: 6.8) with a median 32 (RIC: 26–37), while the mean age of the children was 40.6 months (SD: 10.5) with a median 40 (RIC: 31–50), and 2440 children were male (50.7%). In addition, the mean number of birth order was 2.3 (SD: 1.4), and the mean time of breastfeeding was 19.7 months (SD: 9.2) (Table 1).

**Table 1:** Characteristics of the Peruvian women aged 15–49 years and their children aged 24–59 months included in the study.

**Characteristic**	**Absolute frequency (n = 4738)**	Weighted proportion*
Age of the mother (years)		
Mean (SD)		31.8 (6.8)
Level of education		
No education/ Elementary	943	20.3 (19.0–21.7)
High school	2232	44.5 (42.8–46.3)
College	1563	35.2 (33.4–37.0)
Marital status of the mother		
Single	223	4.5 (3.8–5.4)
Cohabitation with partner	3814	81.7 (80.3–83.0)
Separated/divorced/widowed	701	13.8 (12.6–15.0)
Ethnic self-identification		
Non native	4366	94.2 (93.4–94.9)
Native	372	5.8 (5.1–6.6)
Sex of the child		
Female	2298	49.3 (47.5–51.1)
Male	2440	50.7 (48.9–52.5)
Age of the child (months)		
Mean (SD)		40.6 (10.5)
Undernutrition of the child		
No	4194	88.8 (87.7–89.8)
Yes	544	11.2 (10.2–12.3)
Hemoglobin level of the minor (g/dL)		
Mean (SD)		12.3 (1.3)
Breastfeeding time (months)		
Mean (SD)		19.7 (9.2)
Desired pregnancy		
Yes	2272	48.6 (46.8–50.5)
No	2466	51.4 (49.5–53.2)
Adequate prenatal control		
No	1171	24.8 (23.3–26.5)
Yes	3567	75.2 (73.5–76.7)
Birth order number		
Mean (SD)		2.3 (1.4)
Wealth quintile		
Quintile 1 (richest)	450	13.5 (12.2–14.9)
Quintile 2	639	15.4 (14.0–16.8)
Quintile 3	965	20.2 (18.7–21.7)
Quintile 4	1420	27.0 (25.4–28.7)
Quintile 5 (poorest)	1264	23.9 (22.6–25.4)
Area of residence		
Urban	3379	73.8 (72.5–75.0)
Rural	1359	26.2 (25.0–27.5)
Natural region		
Jungle	1147	17.1 (15.9–18.4)
Highlands	1677	30.7 (29.1–32.5)
Coast	1914	52.2 (50.6–53.7)

SD: standard deviation.

*The weighting factor and sample specifications of the 2019 ENDES were included.

Within the maternal characteristics, it was found that 2232 (44.5%) mothers had high school as their educational level, 3814 mothers (81.7%) lived with a partner, 4366 mothers (94.2%) self-identified as non-native, 3567 mothers (75.2%) had eight or more APCs during the last gestation, and 2466 mothers (51.4%) had an unwanted pregnancy in the last five years. On the other hand, 3379 mothers (73.8%) resided in an urban area, and 1420 mothers (27.0%) belonged to wealth quintile 4. Regarding the characteristics of the children, 544 (11.2%) were malnourished (Table 1).

### 3.2 Maternal depressive symptoms according to maternal, child and sociodemographic characteristics

In relation to the severity of depressive symptoms in the study population, it was found that 178 (3.8%) of women had moderate depressive symptoms, while 95 (2.2%) had severe symptoms. Likewise, it was observed that in both levels of depressive symptoms (moderate and severe), a higher proportion of mothers had high school education (5.4% vs. 2.4%), and were separated, widowed or divorced (6.6% vs. 5.1%) (Table 2).

**Table 2:** Characteristics of Peruvian women aged 15 to 49 years and their children aged 24 to 59 months according to the presence of depression in the mothers and the Patient Health Questionnaire-9.

**Characteristic**	**PHQ-9 0**–**9 (n = 4465)**	**PHQ-9 10**–**14 (n = 178)**	**PHQ_9 15**–**27 (n = 95)**	P value*
Overall	94.0 (93.0–94.9)	3.8 (3.1–4.6)	2.2 (1.6–3.0)	
Age of the mother (years)				
Mean (SD)	31.8 (6.8)	30.6 (6.3)	32.1 (7.3)	0.173
Level of education				
No education/ Elementary	94.1 (91.8–95.8)	3.7 (2.5–5.4)	2.2 (1.2–4.2)	<0.001
High school	92.2 (90.4–93.7)	5.4 (4.2–7.0)	2.4 (1.6–3.5)	
College	96.2 (94.9–97.2)	1.8 (1.2–2.6)	2.0 (1.3–3.1)	
Marital status of the mother				
Single	92.6 (86.0–96.2)	3.3 (1.3–8.0)	4.1 (1.5–10.6)	<0.001
Cohabitation with partner	95.1 (94.0–96.0)	3.3 (2.6–4.2)	1.6 (1.1–2.3)	
Separated/divorced/widowed	88.3 (84.6–91.1)	6.6 (4.7–9.4)	5.1 (3.2–8.1)	
Ethnic self-identification				
Non native	93.9 (92.8–94.9)	3.9 (3.2–4.7)	2.2 (1.6–3.0)	0.625
Native	95.2 (92.3–97.0)	2.8 (1.5–5.2)	2.1 (1.0–4.4)	
Sex of the child				
Female	94.4 (92.7–95.6)	3.2 (2.4–4.3)	2.4 (1.5–3.9)	0.329
Male	93.7 (92.2–94.9)	4.3 (3.3–5.7)	2.0 (1.4–2.8)	
Age of the child (months)				
Mean (SD)	40.7 (10.5)	40.9 (10.2)	39.7 (9.8)	0.792
Undernutrition of the child				
No	94.0 (92.9–94.9)	3.9 (3.2–4.8)	2.1 (1.6–2.7)	0.164
Yes	94.2 (90.7–96.4)	2.6 (1.6–4.2)	3.3 (1.5–6.9)	
Hemoglobin level of the minor (g/dL)				
Mean (SD)	12.3 (1.3)	12.4 (1.3)	12.0 (1.2)	0.190
Breastfeeding time (months)				
Mean (SD)	19.7 (9.0)	20.3 (10.6)	18.5 (11.5)	0.645
Desired pregnancy				
Yes	95.9 (94.6–96.9)	2.7 (1.9–3.8)	1.4 (0.8–2.4)	<0.001
No	92.2 (90.7–93.5)	4.8 (3.8–6.1)	3.0 (2.2–4.0)	
Adequate prenatal control				
No	93.7 (91.8–95.2)	4.4 (3.2–5.9)	1.9 (1.1–3.2)	0.568
Yes	94.1 (92.8–95.2)	3.6 (2.8–4.6)	2.3 (1.6–3.3)	
Birth order number				
Mean (SD)	2.3 (1.4)	2.4 (1.4)	2.6 (1.3)	0.284
Wealth quintile				
Quintile 1 (richest)	95.7 (92.2–97.7)	2.4 (0.9–5.8)	1.9 (0.8–4.3)	0.631
Quintile 2	92.9 (89.3–95.3)	4.6 (2.8–7.4)	2.6 (1.1–5.7)	
Quintile 3	93.6 (91.4–95.3)	4.0 (2.7–6.0)	2.4 (1.5–3.8)	
Quintile 4	92.9 (90.8–94.7)	4.6 (3.3–6.4)	2.4 (1.4–4.0)	
Quintile 5 (poorest)	95.3 (93.7–96.5)	3.0 (2.0–4.3)	1.7 (1.0–2.8)	
Area of residence				
Urban	93.7 (92.3–94.8)	4.0 (3.2–5.0)	2.3 (1.6–3.4)	0.411
Rural	95.0 (93.4–96.2)	3.2 (2.3–4.6)	1.8 (1.1–2.9)	
Natural region				
Jungle	95.1 (93.4–96.4)	3.6 (2.6–5.0)	1.3 (0.7–2.3)	0.496
Highlands	93.8 (92.3–95.0)	4.0 (3.1–5.3)	2.2 (1.5–3.1)	
Coast	93.8 (91.9–95.2)	3.7 (2.7–5.1)	2.5 (1.6–4.0)	

The weighting factor and sample specifications of the 2019 ENDES were included.

* P-value was calculated using the Chi-square test with Rao-Scott correction for categorical variables and Student’s t for continuous variables.

PHQ-9: Patient Health Questionnaire-9; SD: standard deviation.

### 3.3 Self-regulation of emotions and child behaviors according to maternal, child and sociodemographic characteristics

It was found that 3247 children (68.8%) did not self-regulate their emotions and behaviors. The mean age of mothers of children who did not self-regulate emotions and behaviors was 31.6 years (SD: 6.8), most mothers had high school as educational level (70.2%), self-identified as non-native (69.4%), had an unwanted pregnancy in the last five years (72.8%) and less than eight APCs (71.6%) (Table 3).

**Table 3:** Characteristics of Peruvian women aged 15 to 49 years and their children aged 24 to 59 months according to self-regulation emotions and behaviors of the children.

**Characteristic**	**Child does not regulate emotions and behaviors (n = 3247)**	**Child regulates emotions and behaviors (n = 1491)**	P value*
Overall	68.8 (67.1–70.5)	31.2 (29.5–32.9)	
Age of the mother (years)			
Mean (SD)	31.6 (6.8)	32.3 (6.6)	0.009
Level of education			
No education/ Elementary	65.6 (61.9–69.1)	34.4 (30.9–38.1)	0.002
High school	70.2 (67.7–72.6)	29.8 (27.4–32.3)	
College	68.9 (65.7–71.9)	31.1 (28.1–34.3)	
Marital status of the mother			
Single	64.0 (55.4–71.8)	36.0 (28.2–44.6)	0.189
Cohabitation with partner	68.6 (66.6–70.5)	31.4 (29.5–33.4)	
Separated/divorced/widowed	71.9 (67.6–75.8)	28.1 (24.2–32.4)	
Ethnic self-identification			
Non native	69.4 (67.6–71.2)	30.6 (28.8–32.4)	0.001
Native	59.7 (53.6–65.5)	40.3 (34.5–46.4)	
Sex of the child			
Female	68.0 (65.4–70.5)	32.0 (29.5–34.6)	0.332
Male	69.7 (67.3–71.9)	30.3 (28.1–32.7)	
Age of the child (months)			
Mean (SD)	40.0 (10.4)	42.1 (10.4)	<0.001
Undernutrition of the child			
No	69.1 (67.2–70.9)	30.9 (29.1–32.8)	0.428
Yes	67.0 (62.0–71.7)	33.0 (28.3–38.0)	
Hemoglobin level of the minor (g/dL)			
Mean (SD)	12.3 (1.3)	12.4 (1.3)	0.006
Breastfeeding time (months)			
Mean (SD)	19.7 (9.4)	19.6 (8.6)	0.791
Desired pregnancy			
Yes	64.6 (62.0–67.1)	35.4 (32.9–38.0)	<0.001
No	72.8 (70.5–75.0)	27.2 (25.0–29.5)	
Adequate prenatal control			
No	71.6 (68.2–74.9)	28.4 (25.1–31.8)	0.060
Yes	67.9 (65.9–69.9)	32.1 (30.1–34.1)	
Birth order number			
Mean (SD)	2.3 (1.4)	2.4 (1.4)	0.341
Wealth quintile			
Quintile 1 (richest)	69.3 (63.4–74.7)	30.7 (25.3–36.6)	0.008
Quintile 2	71.2 (66.3–75.7)	28.8 (24.3–33.7)	
Quintile 3	72.5 (68.9–75.8)	27.5 (24.2–31.1)	
Quintile 4	69.6 (66.2–72.7)	30.4 (27.3–33.8)	
Quintile 5 (poorest)	63.1 (59.9–66.2)	36.9 (33.8–40.1)	
Area of residence			
Urban	70.3 (68.2–72.4)	29.7 (27.6–31.8)	0.002
Rural	64.7 (61.6–67.6)	35.3 (32.4–38.4)	
Natural region			
Jungle	69.4 (65.8–72.7)	30.6 (27.3–34.2)	<0.001
Highlands	63.4 (60.6–66.1)	36.6 (33.9–39.4)	
Coast	71.8 (69.1–74.4)	28.2 (25.6–30.9)	

The weighting factor and sample specifications of the 2019 ENDES were included.

* P value was calculated using the Chi-square test with Rao-Scott correction correction for categorical variables and Student’s t for continuous variables.

SD: standard deviation.

Regarding the characteristics of the children who did not regulate their emotions and behaviors, the mean age was 40 months (SD: 10.4) and the mean hemoglobin level was 12.3 g/dL (SD: 9.4) (Table 3).

### 3.4 Maternal depression and self-regulation of emotion and behavior in children

A decreasing trend was estimated between children's self-regulation of emotions and behaviors and higher severity of moderate (10–14 points [PR: 0.67; 95% CI: 0.46–0.98]) and severe (15–27 points [PR: 0.48; 95% CI: 0.26–0.86]) maternal depressive symptoms in the crude model. Likewise, in the model adjusted for child characteristics, moderate and severe maternal depression were found to decrease the likelihood of children self-regulating their emotions and behaviors (model 1) (moderate symptoms [10–14 points, aRP: 0. 67; 95% CI: 0.46–0.98) and severe [15–27 points, aRP: 0.48; 95% CI: 0.26–0.86]); however, when adjusting for maternal and household variables, an association was only found with severe depressive symptoms (aRP: 0.48; 95% CI: 0.26–0.86) (model 2) (Table 4).

**Table 4:** Association between maternal depression and self- regulation of emotion and behaviors by the children.

**Characteristic**	**Child does not regulate emotions and behaviors**	**Child regulates emotions and behaviors**	**Crude model**	Model 1*	Model 2**
	**n = 3247**	**n = 1491**	**PR (95% CI)**	**aPR (95% CI)**	**aPR (95% CI)**
Depressive symptoms (PHQ-9)					
0–9	68.1 (66.3–69.8)	31.9 (30.2–33.7)			
10–14	78.6 (69.3–85.7)	21.4 (14.3–30.7)	**0.67 (0.46**–**0.98)**	**0.67 (0.46**–**0.98)**	0.72 (0.49–1.06)
15–27	84.9 (73.7–91.9)	15.1 (8.1–26.3)	**0.47 (0.26**–**0.86)**	**0.48 (0.26**–**0.86)**	**0.48 (0.26**–**0.86)**

* Adjusted for sex of the child, age of the child, undernutrition, hemoglobin level, months of breastfeeding, and adequate prenatal control.

** Adjusted for age of the mother, level of education of the mother, marital status of the mother, ethnic self-identification, sex of the child, age of the child, undernutrition of the child, hemoglobin level, months of breastfeeding, desired pregnancy, adequate prenatal control, birth order number, wealth quintile, area of residence and natural region.

Values in bold are statistically significant (p-value < 0.05).

PR: prevalence ratio; aPR: adjusted prevalence ratio; 95% CI: 95% confidence interval.

## 4 Discussion

The present study was found that seven out of ten children under five years of age do not regulate their emotions or behaviors, while 3.8% of the mothers had moderate depressive symptoms and 2.2% had severe symptoms. Regarding the association of interest, it was found that when adjusting for the characteristics of the children as confounding variables, the children of mothers with moderate and severe depressive symptoms had a lower probability of self-regulating their emotions and behaviors, while when adjusting for the characteristics of the mother and of the dwelling, this association was maintained when the mothers had severe depressive symptoms.

It was found that more than 60% of children under five years of age did not self-regulate their emotions and behaviors, a figure higher than that reported in a study conducted in 63 LMICs, where the prevalence of delay in socioemotional development ranged from 4.6% to 42.4%, with the Central African Republic being one of the countries with the highest prevalence of this delay among LMICs (Gil et al., 2020). In LAC, the prevalence of this disorder ranges between 10.8 and 32.6%, with the highest prevalences observed in Suriname, Guyana and Belize (Gil et al., 2020). Additionally, studies conducted in high-income countries, such as United States (Ghandour et al., 2019) and Canada (Herba et al., 2013), report a lower prevalence of emotional and behavioral problems than that reported in the present study, with 34.6% in American children aged 3 to 5 years and 24.8% in Canadian children aged 5 to 60 months, respectively. The high prevalence of lack of emotions self-regulation in children found in the present study, which is even higher than that reported in most LMICs (including LAC countries) (Gil et al., 2020), could be due to the use of different scales or instruments for the measurement of early child development in developed countries, where the study conducted in Canada was based on an instrument used in the Quebec Longitudinal Study of Child Development, which has items from previously validated instruments such as the Child Behavior Checklist, the Ontario Child Health Study scales, and the Preschool Behavior Questionnaire (Herba et al., 2013), while the study conducted in United States is based on a population-based survey conducted to detect emotional and behavioral disorders in children 3 to 17 years of age (Ghandour et al., 2019). On the other hand, the study conducted in 63 LMICs uses the DHS program format that is similar to the one used in the present study. However, they included a different population (children aged 36 to 59 months) than the one included in the present study and used different questions to construct the variable related to self-regulation of emotions and behaviors (Gil et al., 2020). In this sense, even though this difference may not be comparable with low- and middle- or high-income countries, it exposes a major problem in the child development of Peruvian children.

Likewise, the high prevalence of lack of emotions self-regulation in children found in our study could be attributed to several environmental factors that are more frequently observed in the Peruvian population (compared to other South American countries) and compromise the neurodevelopment of the child. These factors are related to maternal age, child malnutrition due to a lack of exclusive breastfeeding and food supplementation (up to 24 months of age) and low hemoglobin (which conditions less tolerance to frustration), poverty or extreme poverty (which could lead to intergenerational poverty), severe psychosocial deprivation associated with exposure to intimate partner violence against the mother, parental alcohol problems, parental absence, abuse and physical punishment during childhood, and low access to social systems that counteract these traumatic events and residing in rural areas, where there are barriers to accessing social support (Díaz et al., 2017, Saavedra, 2020, Sanchez, 2009, Huicho et al., 2020, Bendini and Dinarte, 2020, Bedoya et al., 2020, Chang et al., 2011). At early ages (<24 months of age), children present the greatest sensitivity to these environmental factors causing cognitive and emotional problems (anxiety, depression, hyperactivity and low self-esteem) in adolescence and adulthood (Black et al., 2017). Therefore, strategies focused on improving all scales of early child development should consider the social and economic factors to which the mother and child are subjected in order to reduce the social, health and economic consequences in adolescence and adulthood.

In relation to the prevalence of maternal depressive symptoms, it was found that 3.8% had moderate depressive symptoms and 2.2% had severe symptoms. These figures are lower than those reported in LAC countries such as Brazil (Bozzini et al., 2021), Argentina (Mathisen et al., 2013), India (Neelakanthi et al., 2021), Nepal (Dawadi et al., 2020), and Vietnam (Van Vo et al., 2017), where higher figures of moderate and severe depressive symptoms were observed in mothers compared to the present study. Although the present study used the PHQ-9 which is a scale previously validated in the Peruvian population to measure depressive symptoms in the last 14 days prior to the survey (Calderón et al., 2012), these differences could be attributed to the use of different scales to measure depressive symptoms in different time periods in mothers, where all the studies included in the comparison used the Edinburgh Postnatal Depression Scale (EPDS) that measures depressive symptoms in the 7 days prior to the survey, which could lead to higher prevalence of depressive symptoms in a short period of time. Additionally, the prevalence of depressive symptoms in Peruvian mothers could be due to several factors that contribute to a higher probability of having mental health disorders, such as unemployment, having an unwanted pregnancy, being single, low socioeconomic status (Macedo-Poma et al., 2019) and lack of medical and psychotherapeutic treatment (Prom et al., 2022), even more so when the population of Peruvian mothers remains without timely diagnosis and lack of access to treatment. These factors limit the interaction between mother and child (including the interruption of breastfeeding), altering the child's development and increasing the probability of having cognitive and emotional problems in the developmental stage (Fenning and Baker, 2012). Therefore, mental health interventions that favor a safe environment for the personal development of the mother and child should be oriented to the socioeconomic factors faced by women, especially during pregnancy and the postpartum period.

The main finding of this study was that, considering child characteristics as confounding variables, children of mothers with moderate and severe depressive symptoms were less likely to regulate their emotions and behaviors, whereas when considering maternal and housing characteristics, the association held when mothers had severe depressive symptoms. Studies in diverse populations in Pakistan, Finland, Canada, United States, Mexico, and Australia reported that maternal depression was negatively associated with children's emotion and behavior regulation (Urizar and Muñoz, 2021, Herba et al., 2013, Conroy et al., 2012, Liu et al., 2017, Tuovinen et al., 2018, Giallo et al., 2018, De Oliveira et al., 2019, Flynn et al., 2017). In contrast to high-income countries, countries with limited economic resources present social and economic factors that could contribute to this association such as low socioeconomic status of the mother at the time of pregnancy that persists until after delivery (leading to food insecurity), poor parental stimulation, intimate partner violence, child abuse and negative social situations that produce an unsafe environment for child and maternal development, making it difficult for adequate self-regulation of children's emotions and behavior (Harris and Santos, 2020, Lovejoy et al., 2000, Palermo et al., 2018). Additionally, biomedical literature indicates that this association has a reverse causality, where emotional and behavioral problems can lead to the development of maternal depressive symptoms. In this regard, several studies report that emotional and behavioral problems in children cause mothers to develop depressive symptoms due to low marital satisfaction, low socioeconomic status that prevents food security for the child, exposure to stressful events such as sleep problems in the child, lack of social support and unfavorable health conditions during pregnancy (Ystrom et al., 2017, van der Waerden J, Galéra C, Saurel-Cubizolles M-J, Sutter-Dallay A-L, Melchior M, the EDEN Mother-Child Cohort Study Group, 2015, Barsisa et al., 2021). Therefore, interventions developed to reduce the impact of depressive symptoms on the lack of self-regulation of emotions and behaviors in children should be focused on reducing the external maternal and child factors that facilitate and strengthen this association.

Among the limitations of the study, there is a possible recall bias. Also, there may be recording errors at the time of data collection. In addition, the cross-sectional design of the study does not allow the evaluation of causal relationships due to a lack of temporality in the measurement of the study variables. Likewise, the relationship between the interest variables has a reverse causality. Since the instrument developed to measure emotion regulation is specific to the Peruvian population, the comparability of our results with other studies would be limited. On the other hand, the use of the PHQ-9 tool to evaluate clinically relevant depressive symptoms may generate the presence of false negatives due to possible response bias. Finally, variables such as birth weight, number of months of pregnancy at delivery, maternal or infant illness, number of children in the same household, food insecurity, social support or mother–child relationship that could explain the relationship between maternal depression and self-regulation of emotions have not been considered. Despite these limitations, the ENDES is the only Peruvian population-based survey conducted by previously trained personnel that assesses depressive symptoms in the population aged 15 years and older and early childhood development in children under five years of age.

In conclusion, approximately 7 out of 10 Peruvian children under five years of age do not self-regulate their emotions and behaviors. Furthermore, children of mothers with moderate and severe depressive symptoms had a lower probability of self-regulating their emotions and behaviors. Therefore, it is necessary to develop maternal education, nutritional and social support programs and mental health strategies from the first level of care aimed at reducing the social, economic and infant factors that may contribute to this association.

### CRediT authorship contribution statement

**Akram Hernández-Vásquez:** Conceptualization, Data curation, Project administration, Formal analysis, Methodology, Visualization, Writing – review & editing. **Rodrigo Vargas-Fernández:** Validation, Writing – original draft, Writing – review & editing. **Fabian Chavez-Ecos:** Validation, Writing – original draft, Writing – review & editing. **Isabel Mendoza-Correa:** Validation, Writing – original draft, Writing – review & editing. **José Del-Carmen-Sara:** Conceptualization, Validation, Writing – original draft, Writing – review & editing.

## Declaration of Competing Interest

The authors declare that they have no known competing financial interests or personal relationships that could have appeared to influence the work reported in this paper.
